# Stress-Mediated Responses of *Aedes aegypti* (Diptera: Culicidae) Larvae When Exposed to *Metarhizium brunneum* (Hypocreales: Clavicipitaceae) and *Toxorhynchites brevipalpis* (Diptera: Culicidae)

**DOI:** 10.1093/jme/tjac110

**Published:** 2022-08-08

**Authors:** Martyn J Wood, Abeer M Alkhaibari, Tariq M Butt

**Affiliations:** Department of Biosciences, Faculty of Science and Engineering, Swansea University, Swansea, UK; Department of Biology, Faculty of Science, University of Tabuk, Tabuk, Kingdom of Saudi Arabia; Department of Biosciences, Faculty of Science and Engineering, Swansea University, Swansea, UK

**Keywords:** *Aedes*, *Toxorhynchites*, *Metarhizium*, caspase, phenoloxidase

## Abstract

*Aedes aegypti* mosquitoes are capable of vectoring a wide range of diseases including dengue, yellow fever, and Zika viruses, with approximately half of the worlds’ population at risk from such diseases. Development of combined predator–parasite treatments for the control of larvae consistently demonstrates increased efficacy over single-agent treatments, however, the mechanism behind the interaction remains unknown. Treatments using the natural predator *Toxorhynchites brevipalpis* and the entomopathogenic fungus *Metarhizium brunneum* were applied in the laboratory against *Ae. aegypti* larvae as both individual and combined treatments to determine the levels of interaction between control strategies. Parallel experiments involved the removal of larvae from test arenas at set intervals during the course of the trial to record whole body caspase and phenoloxidase activities. This was measured via luminometric assay to measure larval stress factors underlying the interactions. Combined *Metarhizium* and *Toxorhynchites* treatments were seen to drastically reduce lethal times as compared to individual treatments. This was accompanied by increased phenoloxidase and caspase activities in combination treatments after 18 h (*p* < 0.001). The sharp increases in caspase and phenoloxidase activities suggest that combined treatments act to increase stress factor responses in the larvae that result in rapid mortality above that of either control agent individually. This work concludes that the underlying mechanism for increased lethality in combined parasite–predator treatments may be related to additive stress factors induced within the target host larvae.

Mosquitoes transmit a wide range of diseases (e.g., chikungunya, dengue, Japanese encephalitis, malaria, Rift Valley fever, West Nile virus, and Zika) that have a devastating impact on human health (Leta et al. 2018). Almost half the world’s population is at risk of mosquito-transmitted diseases with the geographic range expanding due to climate change, trade, and tourism (Ryan et al. 2019, Brugueras et al. 2020, Müller et al. 2020). Mosquito-transmitted diseases such as those listed above, previously considered tropical diseases, are increasingly being reported in Europe (Calzolari et al. 2016, Bakonyi et al. 2020, Brugueras et al. 2020; Tjaden et al. 2021).

Many chemical pesticides used in vector management programs have been withdrawn due to the risks posed to both humans and the environment (Pardío et al. 2012, Hernández et al. 2013, Ruiz-Suárez et al. 2015). The problem of vector management is exacerbated due to the development of insecticide resistance in mosquito populations around the world (Amelia-Yap et al. 2018, Seixas et al. 2017, Gan et al. 2021). Efforts have focused on alternative strategies including use of pathogenic microbes and insect predators (Greenfield et al. 2015; Bruehl et al. 2020; Dambach, 2020). Insect predators of relevance to mosquito control have been identified from numerous orders including, coleoptera, diptera, hemiptera, and odonata (Shaalan et al. 2009). These have the advantage that they can naturally proliferate within the environment, offering potential sustained background control, albeit with a reduced efficacy as compared to insecticidal applications. Of the entomopathogenic biological control agents available, entomopathogenic fungi are of significant interest for commercial development. Several strains of *Metarhizium brunneum* and *M. anisopliae* (Hypocreales: Clavivipitaceae) have been developed that effectively kill a wide range of arthropod hosts, including those of relevance to agro-forestry and vectors of human and animal disease (Scholte et al. 2007, Brunner-Mendoza et al. 2019). Both species show much promise for vector control as they, unlike bacterial pathogens, can infect all developmental stages (adults, eggs, larvae, pupae) of the mosquito (Alkhaibari et al. 2017, Carolino et al. 2019, Choi et al. 2020, de Paula et al. 2021). Another advantage of EPF is that they can work in concert with aquatic natural predators, with the latter usually being more tolerant of *Metarhizium* than *Aedes* larvae (Alkhaibari et al. 2018, Belevich et al. 2018). Recently, it was shown that *M. brunneum* and the natural predator *Toxorhynchites brevipalpis*, applied as simultaneous control agents, significantly increase mortality of *Ae. aegypti* larvae within a shorter time-frame than either agent acting alone (Alkhaibari et al. 2018). The underlying mechanism for this interaction remains to be elucidated, but may be useful in facilitating significantly reduced application requirements for the fungal biocontrol agents, especially given residual natural predator populations within the environment.

Insect stress responses are known to alter immunological pathways through redirection of resources away from immune systems; conserving resources for physiologically demanding activities (Adamo 2014). This effect can increase insect susceptibility to disease, thereby offering a potential route for improved insecticidal control. Earlier work has shown that *M. brunneum* conidia do not adhere to the larval cuticle, rather, the spores are ingested, and the spore bound protease Pr1 induced stress which leads to larval mortality (Butt et al. 2013, Greenfield et al. 2015). This response may in part be explained by the pro-inflammatory state induced by insect stress responses, while ostensibly likely to enhance early immune response (Adamo 2017), this state may facilitate apoptotic cell death, leading to mortality, as described in Butt et al. 2013. Aquatic invertebrate predator species are known to cause a range of direct and indirect effects, including stress amplification in prey species such as mosquito larvae, for which shortened lifespans are an indirect stress-related consequence (Cinel et al. 2020, Alomar and Alto, 2021). Introduction of additional stress factors, in the form of natural predators may, therefore, serve to amplify stress responses of mosquito larvae leading to rapid mortality. This may explain why laboratory application rates of *M. brunneum* can be significantly reduced in the presence of *Tx. brevipalpis*, offering potential cost-reductions relating to entomopathogen applications, alongside significantly reduced nontarget effects (Alkhaibari et al. 2016; Garrido-Jurado et al. 2016). Caspases are excellent stress markers, their levels are elevated in mosquito larvae, and other aquatic invertebrates exposed to EPF and their metabolites (Garrido-Jurado et al. 2016, Butt et al. 2016). Caspases are cysteine proteases that play a pivotal role in regulating inflammation and apoptosis and their levels can be raised before there is manifestation of damage (Butt et al. 2013). Injury triggers several defense reactions, most notable is the activation of prophenoloxidase to its active form phenoloxidase, a key enzyme that leads to the formation of melanin at wound sites and around invading microbes in the hemolymph (Lu et al. 2014; Butt et al. 2016). Melanin will inhibit growth of invading microbes, but its constant production may have fitness costs, reducing egg production, and shortening the mosquito’s lifespan (Ferdig et al. 1993).

The aim of this article was to elucidate upon the mechanisms driving interactions between *M. brunneum* and *Tx. brevipalpis* in relation to increased pathogenicity against *Ae. aegypti* larvae. Improved understanding of these entomopathogen–predator interactions within target species will allow for improved modelling, application, and control strategy design within the natural environment, both for entomopathogenic fungi and natural predator applications

## Methods

### Mosquito Larvae

The eggs of *Ae. aegypti* (strain: AeAe) and *Tx. brevipalpis* were obtained from the London School of Hygiene and Tropical Medicine (LSHTM). *Ae. aegypti* were reared to fourth instar using crushed guinea pig pellets (Pets at Home, UK) in a controlled temperature room at 25 ± 2°C, 65% RH, and a photoperiod of 12:12 (L:D) h. *Tx. brevipalpis* larvae were reared to fourth instar in the same controlled temperature room. These were fed on a separate colony of size appropriate *Ae. aegypti* larvae, while being fed on size appropriate *Ae. Aegypti* larvae; *Ae. aegypti* feed stock was at the same instar as the *Tx. Brevipalpis* larvae.

### Fungal Production

*M. brunneum* isolate ARSEF 4556, identified as an effective pathogen of *Ae. aegypti* (Butt et al. 2013, Greenfield et al. 2015, Alkhaibari et al. 2016) was produced and maintained on broken basmati rice (Ansari and Butt 2011). Conidial viability was determined using the plate count technique after 18 h incubation (Vega et al. 2009). Only batches with viability above 90% were used for further experimentation. The concentration of conidia was determined using an improved Neubauer haemocytometer and diluted to the desired concentration using 0.03% aqueous Tween 80.

### Susceptibility of *Ae. aegypti* and *Tx. brevipalpis* larvae to *M. brunneum*

Larvae of *Tx. brevipalpis* and *Ae. aegypti* were tested for individual susceptibility to *M. brunneum* prior to testing as combined treatments. Protocols were modified from that in Greenfield et al. (2015); 15 *Ae. aegypti* larvae were assessed in 250 ml round plastic tubs (diameter 92 mm) using 150 ml of tap water, 10 *Tx. brevipalpis* larvae were assessed in 1.5 liters. plastic containers (230 × 230 mm) in 1 liter-tap water. Larvae were exposed to three different concentrations of *M. brunneum* (10^5^, 10^6^, 10^7^ conidia ml^−1^) in two formulations; ‘dry conidia’, consisting of a dried spores applied to the surface of the water without adjuvant; and ‘wet conidia’, whereby conidia were suspended in 0.03% (v/v) aqueous Tween 80 (Sigma Aldrich, UK) before being applied at the water’s surface. ‘Dry’ conidial applications will remain at the surface of the water for an extended period, whereas ‘wet’ conidia immediately suspend within the water column. Mortality was recorded each 24 h period for duration of the trials and individuals’ status was determined as ‘dead’ via physiological stimulation. Trials were conducted in triplicate and repeated three times as biological replicates for each of the treatments. All experiments were conducted in a controlled temperature room at 25 ± 2°C, with a photoperiod of 14:10 (L:D) h.

### Exposure of *Ae. aegypti* to Combinations of Both *Tx. brevipalpis* and *M. brunneum*

Bioassays were conducted according to the protocol set for *Aedes* larvae during the initial susceptibility assays within this study. Assays for combined treatment were conducted at concentrations of 1 × 10^5^, 1 × 10^6^, and 1x10^7^ viable conidia mL^-1^, both with and without the introduction of 1 *Tx. brevipalpis* larvae. Control treatments were carried out using 0.03 % aqueous Tween 80 in lieu of conidial suspension, both with and without the addition of a *Toxorhynchites* larva. Mortality was recorded every 24 h throughout the duration of the experiment, with larval status determined via physiological stimulation. *Aedes* larvae that were no longer present within the test arena were counted as consumed by the *Toxorhynchites* larva. All experiments were conducted in a controlled temperature room at 25 ± 2°C, with photoperiod of 14:10 (L:D) h. As with previous assays, all experiments were conducted in triplicate and repeated three times overall.

### The Spatio-chemical Effects of *Tx. brevipalpiss*

Additional assays were conducted to determine whether *Aedes* mortality seen in initial bioassays was influenced by chemicals released by *Toxorhynchites*, or through direct physical exposure; either through feeding related damages inflicted by *Tx. brevipalpis* or through behavioural response to constant predator evasion. For this, 250 ml plastic containers (diameter: 92 mm) were modified with a mesh barrier to separate *Aedes* and *Toxorhynchites* larvae. Experiments were otherwise carried out according to the protocol established in the previous bioassays; using concentrations of 1 × 10^6^, and 1 × 10^7^ viable conidia ml^−1^. Mortality was recorded every 24 h until the conclusion of the experiment, larval status was determined through physiological stimulation. All experiments were conducted in a controlled temperature room at 25 ± 2°C, with a photoperiod of 14:10 (L:D) h. As with previous assays, all experiments were conducted in triplicate and repeated three times overall.

### Effects of *Tx. brevipalpis* Density on the Survival of *Ae. aegypti* Larvae When Treated With *M. brunneum*

Bioassays were also completed to determine the effects *Toxorhynchites* population density may have on *Aedes* mortality using combined applications. In these, larger square plastic tubs (Width 260 mm), each containing 1-liter water and 100 *Aedes* larvae were inoculated with *M. brunneum* at 1 × 10^6^ conidia ml^−1^. 1, 5, 10, or 20 *Toxorhynchites* larvae were introduced in combination with *M. brunneum*. An additional treatment consisting of 1 × 10^6^ conidia ml^−1^ was completed to demonstrate control efficacy using *M. brunneum* only. Control experiments used an application of 0.03% Tween 80 only. Experimental subsets conducted in triplicate were set up according to the reduced Latin square randomization tables described in WHO 2013. Mortality was recorded every 24 h until the conclusion of the experiment, larval status was determined through physiological stimulation. *Aedes* larvae that were no longer present within the test arena were counted as consumed by the *Toxorhynchites* larva. All experiments were conducted in a controlled temperature room at 25 ± 2°C, with a photoperiod of 14:10 (L:D) h. As with previous assays, all experiments were conducted in triplicate and repeated three times overall.

### Caspase Activity

Activity of caspases 2, 3, 7, and 8 was assessed over time in whole body homogenates of *Aedes* larvae (*n* = 10 per treatment) exposed to *M. Metarhizium* and *Tx. brevipalpis* using a BioTek Synergy H1 hybrid reader (Agilent, CA) in three replicate wells for each sample. All samples were exposed to control assays as per methodology described in Section 2.3. Due to rapid mortality, insects were assayed at the lower dose (1 × 10^6^ conidia ml^−1^) and removed for assay at 0, 5, 10, and 18 h post inoculation (PI). Control *Aedes* larvae and those exposed to *M. brunneum* and *Tx. brevipalpis* were also assayed.

*Aedes* larvae were removed from their respective treatments and frozen under liquid nitrogen at each specified time-point before homogenization using a micropestle and mortar. Resuspension of homogenates in 500 μL 0.5% triton lysis buffer (Tris 20 mM, NaCl 100 mM, EDTA 500 mM, 0.5% Triton X-100) was followed by gentle agitation before being incubated on ice for 10 min. Homogenates were centrifuged at 14,000 *g* for 10 min and 35 μL aliquots of the resultant supernatant added to 4 replicate wells in a white walled 96-well microtiter plate (Grenier Bio-one). Luminometric assays for each of Caspases-2, 3/7, and 8 activity were then performed in accordance with manufacturer protocol using Caspase Glo 2, Caspase Glo 3–7, and Caspase Glo 8 assay kits (Promega) through addition of 35 μL of Caspase Glo reagent to each supernatant aliquot. Microtiter plates were agitated gently for 10 s at 180 rpm before incubating at 25°C. Endpoint luminescence was measured after 1 h.

### Phenoloxidase Activity

Phenoloxidase (PO) activity was monitored in total larval homogenates from groups of 15 larvae exposed to combined *Metarhizium* and *Toxorhynchites* treatments as described in Butt et al. 2013. Experiments were conducted, as per the caspase assessments described above, throughout a time course study PI (0 h, 10 min, 20 min, 30 min, 40 min, 50 min, 60 min, 6 h, 12 h, 18h, and 24 h). Larvae were removed at each interval and frozen under liquid nitrogen. 15 Larvae were homogenized in 200 μL of phosphate buffer saline (PBS, pH 7.8) in a 1.5 ml microfuge tube, per replicate. Homogenate was made up to 800 μL with PBS and centrifuged at 3,000 *g* for 20 min. Supernatants were removed and stored at −80°C until required. 5 Replicates were in parallel, the experiment was independently repeated three times. L-DOPA (3, 4-dihydroxy-L-phenyl-alanine, Sigma) was the substrate used to determine PO activity, which was expressed as changes in absorbance/min/mg protein (Dubovskiy et al. 2013). 20 μL of homogenate was added to 180 μL of L-DOPA (4 mg mL^−1^) in a 96-well plate and incubated for 30 min at room temperature. Absorbance was measured at 490 nm at each time point. Homogenate protein contents were determined using Bradford’s method; all assays were made with Biotek Synergy H1 plate reader.

### Statistical Analyses

Survival rates of 1) *Ae. aegypti* and *Tx. brevipalpis* larvae exposed to the different concentrations of *M. brunneum* ARSEF 4556 conidia, 2) *Ae. aegypti* larvae exposed to three concentrations of fungal treatment in presence and absence of *Tx. brevipalpis*, 3) *Ae. aegypti* larvae exposed to *M. brunneum* at 1 × 10^6^ and 1 × 10^7^ conidia ml^−1^ with or without *Tx. brevipalpis* larvae, and 4) *Ae. aegypti* larvae exposed to *M. brunneum* at 1 × 10^6^ conidia ml^−1^ with varied ratios of *Tx. brevipalpis* were analysed using Cox regression, with the resulting hazard ratio values used to assess mortality rates (Greenfield et al. 2015). Survival curves were generated via Kaplan−Meier survival analysis, with pairwise comparisons performed using the log-rank tests (Ansari et al. 2011). LT_50_ and LC_50_ values were estimated for each of the insect species by fitting probit regression models to the quantal response data. Log transformations were applied to the dosage and time independent variables, with an additional parameter fitted to the LC_50_ model to account for the natural mortality rates of *Toxorhynchites* larvae under control conditions. Differences in LT_50_ and LC_50_ were compared by ANOVA and significance levels adjusted where appropriate by application of Tukey’s multiple comparisons post-test. Biochemical data sets were analysed through two-way ANOVA with Bonferroni’s post-hoc -tests. Data sets for both caspase and phenoloxidase analyses were evaluated through two-way ANOVA with Bonferroni’s post-tests. Data were log_10_ transformed to meet ANOVA assumptions of homogeneity of variance. All analyses were carried out using SPSS v22.0 (IMB Corporation, USA) and GraphPad Prism v5.0 (Graphpad Software, USA).

## Results

### Susceptibility of *Ae. aegypti* and *Tx. brevipalpis* Larvae to *M. brunneum*

**Fig. 1.**
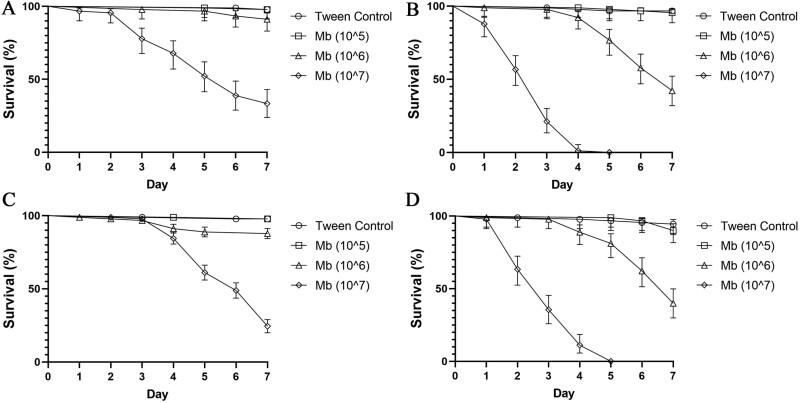
(A−D): Survival curves of *Aedes aegypti* and *Toxorhynchites brevipalpis* exposed to different concentrations of ‘wet and dry’ formulated *Metarhizium brunneum* conidia (ARSEF 4556). Percentage cumulative survival of *Tx. brevipalpis* exposed ‘wet’ (A) and ‘dry’ (C) conidia and *Ae. aegypti* exposed to the same ‘wet’ (B) and ‘dry’ (D) formulations of *M. brunneum* using three different concentrations (10^5^, 10^6^, 10^7^ conidia ml^−1^) for 7 d. Controls were exposed to Tween 80 (0.03%) only. Error is represented as 95% CI. Key: Mb – *Metarhizium brunneum*.

Both *Ae. aegypti* and *Tx. brevipalpis* were susceptible to infection with both wet and dry *M. brunneum* conidia (ARSEF 4556). In both wet and dry applications, *Aedes* mortality was significantly higher than control samples at 1 × 10^6^ conidia ml^−1^ and 1 × 10^7^ conidia ml^−1^ (*p* < 0.001). Applications at 1 × 10^5^ conidia ml^−1^ did not cause mortality significantly above that of the control samples (*p* = 0.189) (Fig. 1B and D). *Toxorhynchites* larvae demonstrate similar susceptibilities (Fig. 1A and C) excepting that no significant mortality was observed for dry applications at 1 × 10^6^ conidia ml^−1^ (Fig. 1C; Table 1). No significant differences in LT_50_ were observed between ‘wet’ and ‘dry’ spore application (Table 2) for either *Tx. brevipalpis* or *Ae. aegypti*. Between the two species, however, *Tx. brevipalpis* was seen to have a lower susceptibility to *M. brunneum* application, demonstrated by the higher LT_50_ values recorded as compared to *Ae. aegypti* larvae (Table 2).

**Table 1.** Statistical significance of Kaplan–Meier log-rank pairwise comparisons *for Aedes aegypti* larvae and *Toxorhynchites brevipalpis* larvae treated with three concentrations of *Metarhizium brunneum* conidia

	Formulation	Wet				Dry			
	Concentration	Control	10^5^	10^6^	10^7^	Control	10^5^	10^6^	10^7^
*Toxorhynchites*	10^5^	ns		*	***	ns		*	***
	10^6^	*	*		***	ns	***		***
	10^7^	***	***	***		***	***	***	
*Aedes*	10^5^	ns		***	***	ns		***	***
	10^6^	***	***		***	***	***		***
	10^7^	***	***	***		***	***	***	

Statistical significance levels derived from post-hoc Tukey’s multiple comparisons analysis for each treatment. *Tx. brevipalpis* and *Ae. aegypti* were individually screened for susceptibility to *M. brunneum* at each of three concentrations (1 × 10^5^, 10^6^, and 10^7^ conidia ml^−1^) Controls consist of Tween 80 (0.03%) application only. ns = not significant, * = *p* < 0.05, ** = p < 0.01, *** = p < 0.001.

**Table 2.** LT_50_ and LC_50_ values estimated for *Aedes aegypti* and *Toxorhynchites brevipalpis* larvae when treated with *Metarhizium brunneum* (ARSEF 4556)

		*Tx. brevipalpis*	*Ae. aegypti*
Wet Conidia	LT_50_	15.57 d (10.26–28.09)	7.58 d (5.93–10.42)
	LC_50_	7.82 × 10^6^ (2.84 × 10^6^–2.19 × 10^7^)	5.92 × 10^5^ (3.41 × 10^5^–2 × 10^6^)
Dry Conidia	LT_50_	11.91 d (10.32–13.94)	7.68 d (6.3–9.49)
	LC_50_	7.18 × 10^6^ (9.89 × 10^5^–2.06 × 10^7^)	9.42 × 10^5^(1.42 × 10^5^–2.14 × 10^6^)

Mean lethal time (LT_50_) for each of ‘wet’ and ‘dry’ *M. brunneum* conidial formulations against *Ae. aegypti* and *Tx. brevipalpis* at a concentration of 1 × 10^6^ conidia ml^−1^. Mean lethal dose (LC_50_) for both ‘wet’ and ‘dry’ conidia formulations against each species at day 7. The 95% confidence intervals are given in parentheses.

In both *Ae. aegypti* and *Tx. brevipalpis*, no significant differences in LC_50_ values were recorded between wet and dry conidial applications (Table 2). In wet applications, fungal applications resulted in higher LC_50_ values for *Tx. brevipalpis* than *Ae. aegypti*, dry applications produced no significant differences between the LC_50_ values for *Tx. brevipalpis* and *Ae. aegypti*.

### *Ae. aegypti* Survival Postexposure to Fungal Conidia With or Without *Toxorhynchites* Larva

**Fig. 2.**
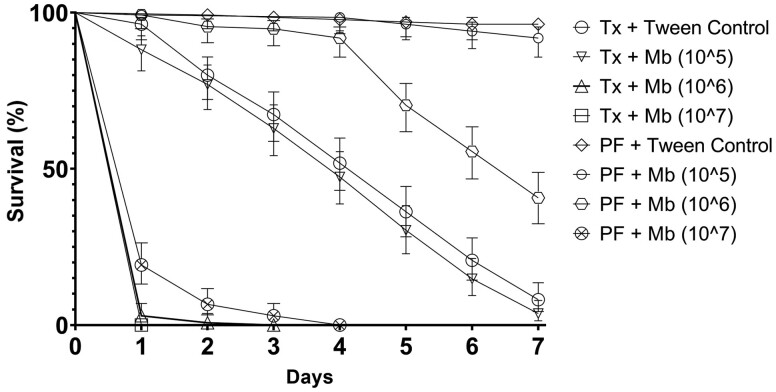
Survival of *Aedes aegypti* larvae when treated with *Metarhizium brunneum* either with or without the addition of *Toxorhynchites brevipalpis.* Cumulative survival (%) of *Ae. aegypti* exposed to three concentrations of *M. brunneum* (10^5^, 10^6^, 10^7^ conidia ml^−1^) using ‘wet’ formulations; either with or without the inclusion of one *Tx. brevipalpis* larva for 7 d. Controls were exposed to Tween 80 (0.03%) application only. Error is represented as 95% CI. Key: Mb – *Metarhizium brunneum*; Tx – *Toxorhynchites brevipalpis*; concentration given in parenthesis – spores ml^−1^.

LT_50_ values were estimated for all assays (Table 3). Significant differences were found between all treatments except *Ae. aegypti* treated with 1 × 10^5^ conidia ml^−1^ either with or without the addition of *Tx. brevipalpis* larvae (Fig. 2). *Ae. aegypti* larvae were highly susceptible to *M. brunneum* at concentrations of 1 × 10^6^ conidia ml^−1^ and 1 × 10^7^ conidia ml^−1^. In both cases mortality was significantly higher in larvae exposed to *M. brunneum* than in untreated controls at (*p* < 0.01). Likewise, *Ae. aegypti* larvae exposed to a *Tx. brevipalpis* larva suffered significant mortality as compared to controls, reaching 90% mortality after 168 h (*p* < 0.001). The rate of *Aedes* mortality caused by *Tx. brevipalpis* was similar to that of *M. brunneum* treatments of 1 × 10^6^ and 1 × 10^7^ conidia ml^−1^ (*p* = 0.187 & *p* = 0.678 respectively). When *Ae. aegypti* larvae were treated with combinations of *M. brunneum* and *Tx. brevipalpis*; mean survival times were significantly lower as compared other treatments and controls (*p* < 0.001). Combined treatments with fungal concentrations of 1 × 10^6^ and 1 × 10^7^ conidia ml^−1^ reached 96.3% and 100% mortality, respectively, after just 24 h. Treatments consisting of *Tx. brevipalpis* larvae applied in combination with *M. brunneum* at 1 × 10^5^ conidia ml^−1^ resulted in *Aedes* mortality at similar levels to treatments consisting of *Tx. brevipalpis* additions only (*p* = 1.000), however, both of these resulted in significantly higher mortality than control experiments (*p* < 0.001).

**Table 3.** Average LT_50_ estimates for *Aedes aegypti* larvae treated with *Metarhizium brunneum* both with and without the addition of *Toxorhynchites brevipalpis larvae*

Without *Tx. brevipalpis*				With *Tx. brevipalpis*			
Control	10^5^	10^6^	10^7^	Control	10^5^	10^6^	10^7^
22.087 (15.688–33.440)	22.130 (15.688–33.440)	8.130 (6.926–10.266)	0.533 (0.367–0.729)	3.677 (3.154–4.284)	3.058 (2.611–3.577)	0.358 (0.218–0.547)	0.294 (0.164–0.487)

Median lethal time (LT_50_) for *Ae. aegypti* larvae treated with *M. brunneum* at three different concentrations (1 × 10^5^, 10^6^, and 10^7^ conidia ml^−1^), both with and without the addition of one *Tx. brevipalpis* larva within the test arena For *Aedes* larvae exposed to *Metarhizium* only, control experiments were exposed to Tween 80 (0.03%) only. For *Aedes* larvae exposed to both *Metarhizium* and *Toxorhynchites* larvae, controls were exposed to a *Toxorhynchites* larva and Tween 80 (0.03%) only. The 95% confidence intervals are given in parentheses.

### Assessment of the Spatio-chemical Effects of *Tx. brevipalpis* Larvae on *Ae. aegypti* Survival

**Fig. 3.**
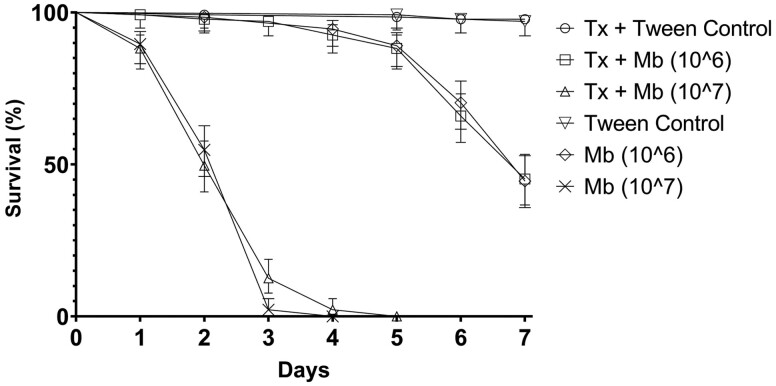
Survival of *Aedes aegypti* larvae when treated with *Metarhizium brunneum* either with or without the addition of a *Toxorhynchites brevipalpis* larvae that was physically separated from the *Aedes* larvae within the test arena. Mean survival (%) of *Ae. aegypti* exposed to two concentrations (10^6^ and 10^7^ conidia ml^−1^) of ‘wet’ formulated *M. brunneum* (ARSEF 4556) both with and without the presence of a *Tx. brevipalpis* larva that had been physically separated from the *Ae. aegypti* larvae in the test arena via a mesh curtain. Controls were exposed to Tween 80 (0.03%) applications only. Error is represented as 95% CI. Key: Mb – *Metarhizium brunneum*; Tx – *Toxorhynchites brevipalpis*.

During experiments in which *Toxorhynchites* larvae were physically separated from the *Aedes* larvae, no spatio-chemical effects were observed that would suggest chemical markers were responsible for increased lethality in combined treatments (Fig. 3). Treatment efficacy was seen to be similar for those *Aedes* larvae exposed to combined *Toxorhynchites* and *Metarhizium* treatments and those *Aedes* larvae that were treated with *Metarhizium* only *(p <* 0.001). No significant difference in LT_50_ values was recorded for combined or individual treatments at either 1 × 10^6^ conidia ml^−1^ (F_(2,18)_ = 2.043 *p* = 0.893) or 1 × 10^7^ conidia ml^−1^ (F_(2,18)_ = 3.019 *p* = 0.657). All combined treatments were significantly different to untreated controls (*p* < 0.001) and controls with 1 physically separated *Toxorhynchites* larva (*p* < 0.001).

During experiments in which the *Toxorhynchites* were physically separated from the *Aedes* larvae, mortality rates were the same for those *Aedes* larvae exposed to combined *Toxorhynchites* (without barrier) and *Metarhizium* treatments, and those *Aedes* larvae that were treated with *Metarhizium* with one physically separated *Toxorhynchites* larva (*p* < 0.001).

No significant differences were recorded for combined treatments with and without one physically separated *Toxorhynchites* larva at either 1 × 10^6^ conidia ml^−1^ (F_(2,18)_ = 2.043 *p* = 0.893) or 1 × 10^7^ conidia ml^−1^ (F_(2,18)_= 3.019 *p* = 0.657). All combined treatments were significantly different to untreated controls (*p* < 0.001) and controls with 1 physically separated *Toxorhynchites* larva (*p* < 0.001).

### Effect of *Tx. brevipalpis* Density on *Aedes* Larvae Survival in Treatments

**Fig. 4.**
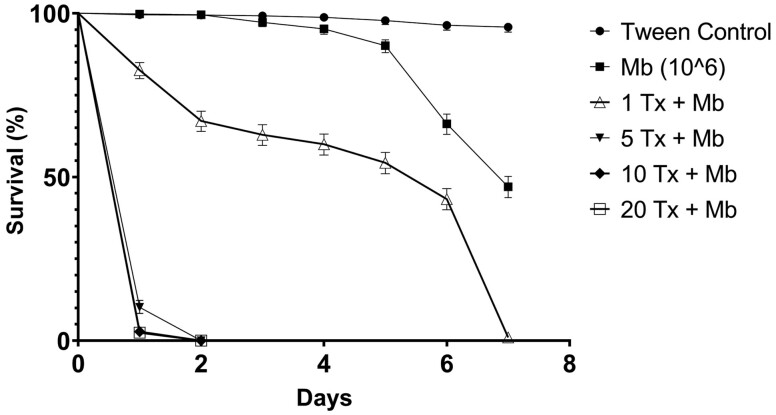
Survival of *Aedes aegypti* treated with *Metarhizium brunneum* combined with different *Tx. brevipalpis* densities per treatment. Mean survival (%) of *Ae. aegypti* larvae exposed to ‘wet’ formulated *Metarhizium brunneum* (1 × 10^6^ conidia ml^−1^) combined with 1, 5, 10, or 20 *Tx. brevipalpis* larvae over a period of 7 d. Controls were exposed to Tween 80 (0.03%) application only. Error is represented as 95% CI. Key: Mb – *Metarhizium brunneum*; Tx – *Toxorhynchites brevipalpis*.

*Toxorhynchites* density, relative to *Aedes* abundance, was seen to have an impact on survival (Fig. 4). Treatment densities comprising 1(*Tx*):10(*Ae*) larvae resulted in similar mortality levels to those recorded during previous experiments. 97.8% mortality was recorded within 24 h, significantly higher than either untreated controls or those treated solely with 1 × 10^6^ conidia ml^−1^ (*p* < 0.001). When LT_50_ estimates were compared between treatment groups, *Toxorhynchites*: *Aedes* densities of 1:5, 1:10, & 1:20 all produced LT_50_ values significantly lower than the *Metarhizium* only positive controls (F_(5,45)_ = 44.598 *p* = <0.001). Only a ratio of 1(*Tx*):100(*Ae*) resulted in a significantly higher LT_50_ as compared to other treatment densities, although in comparison to the *Metarhizium* only treatments, the LT_50_ value remained significantly lower (*p* < 0.001). Cannibalism amongst *Tx. brevipalpis* was only seen to occur after consumption of all *Ae. aegypti* larvae present in treatments.

### Caspase Activity

**Fig. 5.**
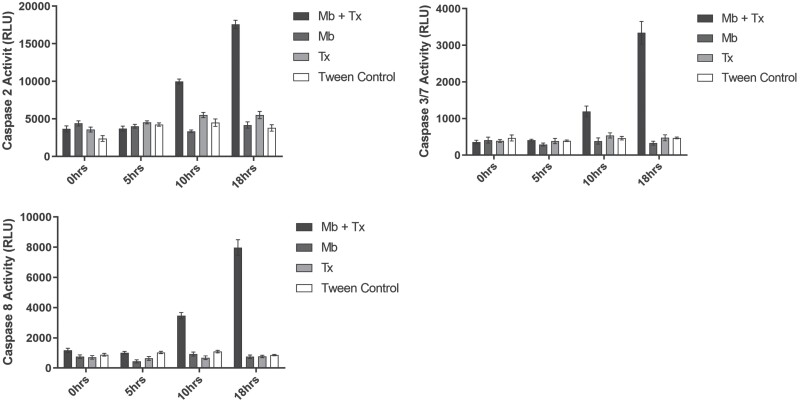
(A–C): Caspase activity for caspases 2 (A), 3/7 (B), and 8 (C) in total body homogenates of *Aedes aegypti* larvae exposed to *Metarhizium brunneum* and *Toxorhynchites brevipalpis*. Caspase activities in *Ae. aegypti* larvae under treatment conditions utilizing *Tx. brevipalpis* and *M. brunneum* as individual and combined treatments. ‘Wet’ formulated *M. brunneum* was applied at a concentration of 1 × 10^6^ conidia ml^−1^; treatments utilizing *Tx. brevipalpis* used one larva per replicate. Controls were exposed to Tween 80 (0.03%) applications only. Error is represented as 95% CI. Key: Mb – *Metarhizium brunneum*; Tx – *Toxorhynchites brevipalpis*.

Overall caspase activity for caspases 2, 3/7, and 8 was consistently higher in *Ae. aegypti* larvae exposed to combined *M. brunneum* and *Tx. brevipalpis* treatments (F_(3,81)_ = 343.15, *p* < 0.001). These effects were seen to be most prominent for caspases 3/7 and 8 (Fig. 5A–C). Groups individually treated with *M. brunneum* or *Tx. brevipalpis* larvae did not express caspase activities significantly different to those in control assays (*p* = 0.994 and *p* = 0.068 respectively). Within the treatment groups, *Ae. aegypti* larvae exposed to *Toxorhynchites* as a sole control agent demonstrated the lowest caspase activity response after 18 h, followed by those treated with *M. brunneum* only.

### Phenoloxidase Activity

**Fig. 6.**
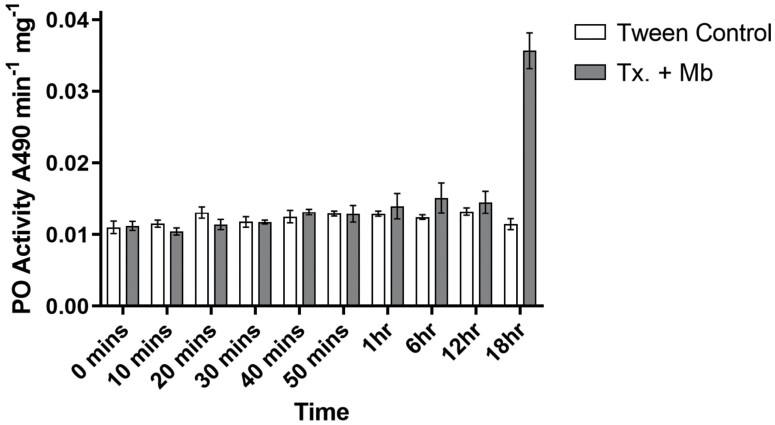
Phenoloxidase activity associated with assays combining *Metarhizium brunneum* and *Toxorhynchites brevipalpis* over time. Phenoloxidase activity, as an indicator of phenoloxidase cascade initiation, in total body homogenates of *Ae. aegypti* larvae after exposure to *M. brunneum* (1 × 10^6^ conidia ml^−1^) in the presence of one *Tx. brevipalpis* larva. Controls were exposed to Tween 80 (0.03%) applications only. Error is represented as 95% CI. Key: Mb – *Metarhizium brunneum*; Tx – *Toxorhynchites brevipalpis*.

Phenoloxidase activity was not significantly modified by the combined treatments including *M. brunneum* and *Tx. brevipalpis* over the course of the experiment until 18 h PI, concurrent with death (F_(3,81)_ = 168.241, *p* = <0.001). In individual treatments, no significant differences were detected in comparison to control samples (Fig. 6).

## Discussion

The results of these experiments elucidate upon the interactions noted between *Tx. brevipalpis* and *M. brunneum*, whereby mortality in *Ae. aegypti* larvae has been seen to increase rapidly. Individual applications of *Tx. brevipalpis* and *M. brunneum* result in significant degrees of mortality in treated *Ae. aegypti* groups, this effect is exacerbated to a significant degree when combination treatments are applied. These combination treatments elicit a clear caspase and phenoloxidase cascade, implying predator-associated stress factors are key components in the control agent interaction.

While both species were seen to be susceptible to *M. brunneum*, ‘wet’ applications were seen to affect *Tx. brevipalpis* to a lesser degree than ‘dry’ spore inoculation. Counter to this, the same ‘wet’ applications were seen to elicit a greater response in *Ae. aegypti* larvae. This is likely due to behavior, *Toxorhynchites* species predominantly spend their time at the water surface, where they will ambush nearby larvae of a similar size. *Aedes* larvae, by contrast, are preferential substrate feeders, often moving down in the water column to feed off of settled particulate matter (Merritt et al. 1992). Given that wet applications of *M. brunneum* sediment quickly, much of the available inoculum would be removed from *Toxorhynchites* preferential level within the water column, simultaneously, this inoculum would readily aggregate in preferential feeding areas for *Aedes* larvae. Such considerations to formulation, therefore, must be considered in systems whereby surface dwelling beneficials are present in treatment areas.

The mode of pathogenesis of *Metarhizium* in terrestrial applications is well understood to be through cuticular invasion followed by colonisation of the haemocoel (Butt et al. 2016) alongside fungal metabolite induced pathogenic activities such as destruxin release (Crisan 1971, Lacey et al. 1988). It had been assumed that the same processes were also the primary mode of pathogenesis in the aquatic environment (Riba et al. 1986, Lacey et al. 1988, and Bukhari et al. 2010), however, Butt et al. (2013) demonstrated mortality can be largely mediated through protease induced stress, leading to apoptotic cell death, and eventually organismal death. While previous studies have focused on increasing fungal pathogenicity (Daoust and Roberts 1982, Shah et al. 2005, and Wang and St. Leger 2007) and viability (Daoust and Roberts 1983), few, if any, have focused on increasing external stress factors. Predator−prey interactions often dominate confined aquatic environments, with organisms under environmental stress shown to be less long-lived, smaller, and often less successful reproductively (Alomar and Alto, 2021). By creating a ‘landscape of fear’, predators could potentially be used to influence prey behavior and physiology, thereby increasing biological control potential and reducing vector capacity. This study shows for the first time that stress factors within the aquatic environment can be increased using predatory species, resulting in increased lethality beyond the capabilities of a single control agent such as *M. brunneum.*

Mosquito larvae have been seen to be effectively controlled with several strains of *M. brunneum* as singular agent (Bukhari et al. 2010), albeit with significant differences in control rates based on strain and formulation (Greenfield et al. 2015, Alkhaibari et al. 2017). Such methods require relatively high concentrations of conidia to be applied to cause 100% mortality in less than 48 h. The mortality enhancing effects seen in combined *Metarhizium* and *Toxorhynchites* treatment on *Aedes* larvae corroborates the work of Alkhaibari et al. (2018), while adding to the growing list of efficacy enhancing interactions reported between entomopathogenic fungi and other biocontrol agents including bacteria, entomopathogenic nematodes, and predatory mites (Ansari et al. 2008, Dogan et al. 2017, Imperiali et al. 2017, Wakil et al. 2017). Such effects have the potential to not only reduce biological control application requirements, but also offer a chemical-free approach to such techniques.

Although it is generally accepted that stress can increase the host’s susceptibility to infection the biochemical pathways have not been well elucidated (Butt et al. 2016). It is tempting to speculate that the apoptotic responses in *Aedes* larvae, previously described in association with *Metarhizium*, may have been exacerbated by presence of the *Tx. brevipalpis* larvae, resulting in rapid mortality with significantly lower concentrations of the fungus. Indeed, the caspase assays support this hypothesis. Both initiator (caspases 2 and 8) and effector (caspases 3 and 7) caspase activity increased with time, suggesting a continued increase in stress response that could lead to exacerbated apoptotic cell death as per Butt et al. (2013), eventually leading to death.

The efficacy enhancing interactions between *M. brunneum* and *Tx. brevipalpis* have the potential to not only reduce conidial application requirements, but have been shown to significantly accelerate *Aedes* mortality under laboratory conditions. The experiments described herein demonstrate temporal larvicidal efficacy comparable with, or superior to, conventional chemical pesticides and the biopesticide *Bacillus thurungiensis israelensis* (Bti) (Bacillales: Bacillaceae) (Kovendan et al. 2011, Mansour et al. 2012, and Araújo et al. 2013). Given that widespread adoption of *Metarhizium* usage is dependent on the length of time required to kill target invertebrates, increases in mortal rapidity produced through control agent interactions have the potential to improve the outlook for future adoption. Furthermore, *M. brunneum* and *Tx. brevipalpis* offer an alternative to Bti and chemical insecticides to which *Ae. aegypti* and other mosquito vectors have developed resistance (Gan et al. 2021), while reduced application requirements for *M. brunneum* will reduce the risks to nontarget aquatic invertebrates (Garrido-Jurado et al. 2016).

Although both *Metarhizium* and *Toxorhynchites* species could work alone, the combination treatments have many benefits, as outlined above. The limitation is the predator, application of these organisms would be less easy than that of *Metarhizium* pesticide spraying of environments. Due to the persistence of *Toxorhynchites* species, and other predators, within the natural environments of *Aedes* larvae, naturalized populations may reduce the requirements for direct human intervention. Furthermore, human assisted introduction of *Toxorhynchites* larvae may result in over-abundance of the predators, which are known to become cannibalistic at high densities (Donald et al. 2020, Collins and Blackwell, 2000). Through reliance on natural populations, these effects may be mitigated to some extent, while data accumulated about natural localized populations could be used in tandem with information presented herein to reduce application requirements for *Metarhizium*.
